# The case of infected intra-abdominal hematoma complicating endoscopic ultrasound-guided tissue acquisition

**DOI:** 10.1055/a-2285-2401

**Published:** 2024-04-09

**Authors:** Daiki Agarie, Susumu Hijioka, Yoshikuni Nagashio, Yuta Maruki, Mark Chatto, Yutaka Saito, Takuji Okusaka

**Affiliations:** 1 68380 Department of Hepatobiliary and Pancreatic Oncology, National Cancer Center Hospital, Tokyo, Japan; 2 37571 Department of Medicine, Makati Medical Center, Manila, Philippines; 3 68380 Endoscopy Division, National Cancer Center Hospital, Tokyo, Japan

Endoscopic ultrasound-guided tissue acquisition (EUS-TA) rarely causes complications. Severe bleeding requiring blood transfusion or hemostasis has occurred only in 0.05–0.2% of cases 1 2 3 . Herein we report, to the best of our knowledge, the first case of EUS-TA complicated by an infected intra-abdominal hematoma ( Video 1 ).

An infected intra-abdominal hematoma complicating endoscopic ultrasound-guided tissue acquisition.Video 1

An 81-year-old man presented with a pancreatic tail tumor on contrast-enhanced computed tomography (CECT) ( Fig. 1 **a** ). EUS-TA was performed using a 22-gauge Franseen needle (Sonotip TopGain; Medi-Globe, Rohrdorf, Germany). After puncture, little fluid accumulated around the tumor, and bleeding into the stomach was observed ( Fig. 1 **b** , **c** ). Scope balloon compression was performed, and hemostasis was achieved ( Fig. 1 **d** ). The following day, gastrointestinal endoscopy was performed to confirm that hemostasis was maintained, and the patient was discharged. Five days after EUS-TA, the patient was rehospitalized because of fever and abdominal pain. Computed tomography (CT) revealed an intra-abdominal hematoma ( Fig. 2 **a** ) and a pseudoaneurysm in a branch of the left gastric artery ( Fig. 2 **b** ). Antibiotic treatment was initiated, followed by transcatheter arterial embolization ( Fig. 2 **c** , **d** ). The symptoms improved; however, C-reactive protein level elevated to 17.7 mg/dL 12 days after EUS-TA. Therefore, EUS-guided cyst drainage was performed and two plastic stents and a nasal drainage catheter were inserted ( Fig. 3 ). Hematoma cultures revealed an *Enterococcus faecalis* infection. CT performed on the day after the cyst drainage showed hematoma shrinkage ( Fig. 4 **a** ).

**Fig. 1:**
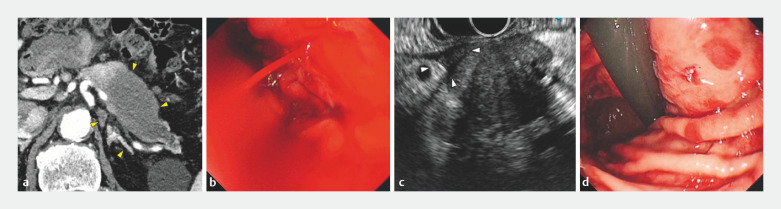
Bleeding and hemostasis during endoscopic ultrasound-guided tissue acquisition (EUS-TA). **a** 52 × 31-mm mass was observed in the pancreatic tail (yellow arrowheads). **b** Bleeding into the stomach was observed after EUS-TA. **c** A small amount of fluid around the tumor (white arrowheads). **d** Hemostasis confirmed after scope balloon compression (white arrows).

**Fig. 2:**
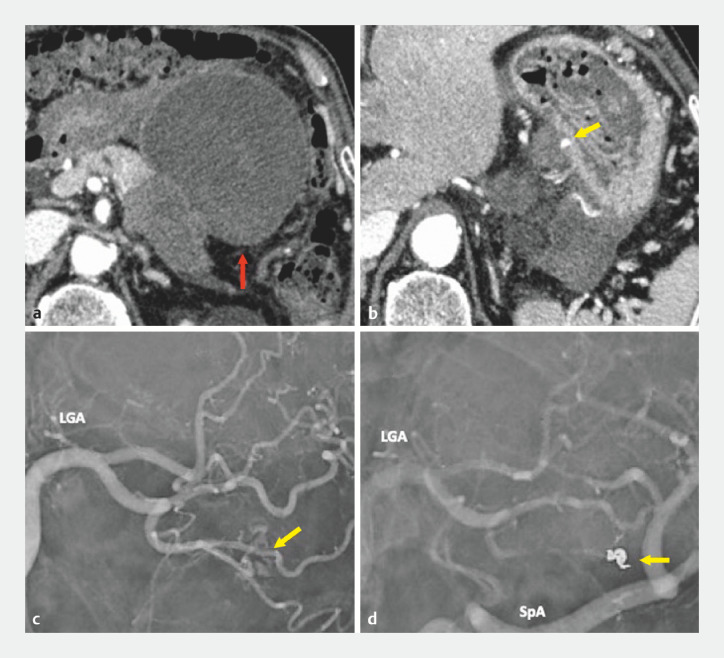
Contrast-enhanced computed tomography (CECT) image of hematoma and emergency transcatheter arterial embolization (TAE) image. **a** CECT showing a hematoma 80 mm in diameter (red arrow). **b** Pseudoaneurysm in the left gastric artery branch (yellow arrow). **c,d** TAE was performed targeting the pseudoaneurysm (yellow arrow).

**Fig. 3:**
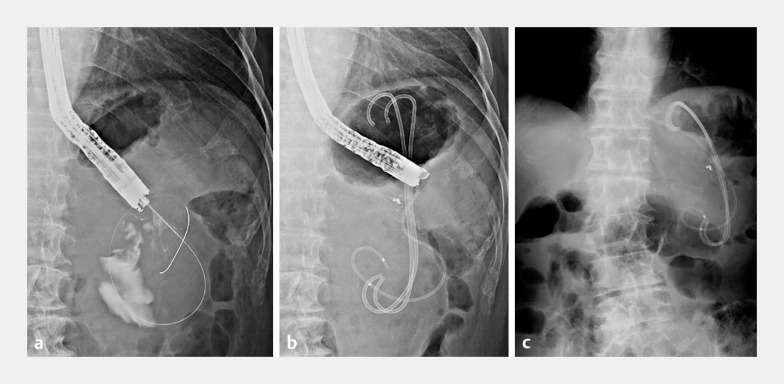
Endoscopic ultrasound-guided cyst drainage was performed for an infected hematoma with insertion of two plastic stents and a nasal drainage catheter.

**Fig. 4:**
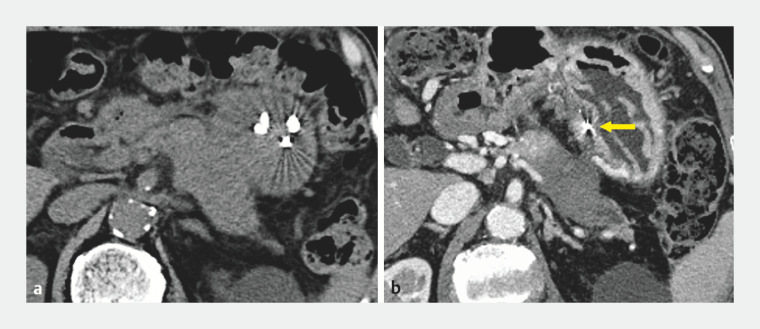
CECT images of the hematoma after cyst drainage. **a** CT scan on the day after the cyst drainage showing shrinkage of the hematoma. **b** CECT two months later showing complete disappearance of the hematoma. Yellow arrows indicate the coil embolization area.

The pancreatic tumor was diagnosed as an adenocarcinoma and neoadjuvant chemotherapy was initiated 12 days after EUS-guided cyst drainage. Two months later, CT confirmed the disappearance of the hematoma ( Fig. 4 **b** ). Three months later, a pancreatic tail resection was performed. Although slight adhesions were observed, surgery was completed without complications. The patient is recurrence-free for one year postoperatively.

A retrospective review of the EUS-TA video identified a small vascular structure within the puncture pathway. Even vessels of small diameter are susceptible to hemorrhage; therefore, the puncture pathway should be carefully observed.

Endoscopy_UCTN_Code_TTT_1AS_2AF

## Notes

Endoscopy E-Videos https://eref.thieme.de/e-videos
*E-Videos* is an open access online section of the journal *Endoscopy* , reporting on interesting cases and new techniques in gastroenterological endoscopy. All papers include a high-quality video and are published with a Creative Commons CC-BY license. Endoscopy E-Videos qualify for HINARI discounts and waivers and eligibility is automatically checked during the submission process. We grant 100% waivers to articles whose corresponding authors are based in Group A countries and 50% waivers to those who are based in Group B countries as classified by Research4Life (see: https://www.research4life.org/access/eligibility/ ). This section has its own submission website at https://mc.manuscriptcentral.com/e-videos .
